# Supplementary material for: Complex‐centric proteome profiling by SEC‐SWATH‐MS
Source: Mol Syst Biol. 2019 Jan 14;15(1):e8438. doi: 10.15252/msb.20188438 (PMC6346213; doi:10.15252/msb.20188438)
Supplement: Supplementary file 8 — Dataset EV7 [file MSB-15-e8438-s008.zip › feature_plots_string/P62714.pdf]

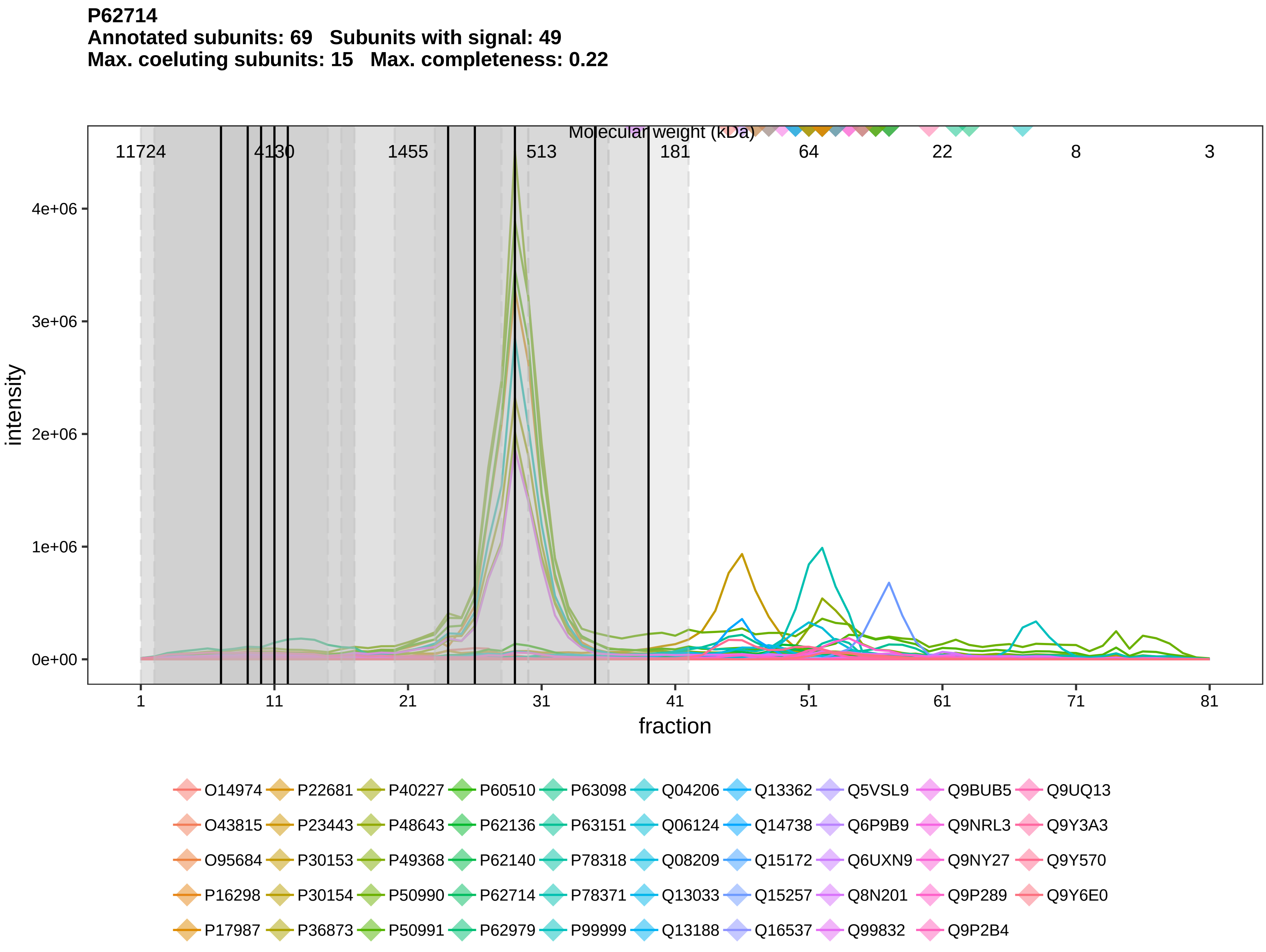

P62714
Annotated subunits: 69 Subunits with signal: 49
Max. coeluting subunits: 15 Max. completeness: 0.22
Molecular weight (kDa)
11724
4130
1455
513
181
64
22
8
3
intensity
4e+06
3e+06
2e+06
1e+06
0e+00
1
11
21
31
41
51
61
71
81
fraction
O14974
P22681
P40227
P60510
P63098
Q04206
Q13362
Q5VSL9
Q9BUB5
Q9UQ13
O43815
P23443
P48643
P62136
P63151
Q06124
Q14738
Q6P9B9
Q9NRL3
Q9Y3A3
O95684
P30153
P49368
P62140
P78318
Q08209
Q15172
Q6UXN9
Q9NY27
Q9Y570
P16298
P30154
P50990
P62714
P78371
Q13033
Q15257
Q8N201
Q9P289
Q9Y6E0
P17987
P36873
P50991
P62979
P99999
Q13188
Q16537
Q99832
Q9P2B4
